# Epidemiology of limb reduction defects as registered in the Medical Birth Registry of Norway, 1970-2016: Population based study

**DOI:** 10.1371/journal.pone.0219930

**Published:** 2019-07-17

**Authors:** Kari Klungsøyr, Tone Irene Nordtveit, Trine Sand Kaastad, Sigrun Solberg, Ida Neergård Sletten, Anne-Karin Vik

**Affiliations:** 1 Department of Global Public Health and Primary Care, University of Bergen, Bergen, Norway; 2 Division of Mental and Physical Health, Norwegian Institute of Public Health, Bergen, Norway; 3 Department of Medical Genetics, Haukeland University Hospital, Bergen, Norway; 4 Division of Orthopaedic Surgery, Oslo University Hospital, Oslo, Norway; 5 Department for Quality and Patient Safety, Oslo University Hospital, Oslo, Norway; 6 Orthopaedic Clinic, Haukeland University Hospital, Bergen, Norway; 7 National Professional Network for Dysmelia, Norwegian National Advisory Unit on Rare Disorders TRS, Oslo, Norway; Centre Hospitalier Universitaire Vaudois, FRANCE

## Abstract

**Background:**

Following the Thalidomide disaster, the Medical Birth Registry of Norway (MBRN) was established in 1967, with epidemiological surveillance of congenital anomalies as one main aim. Limb reduction defects (LRD) constitute a rare and heterogeneous anomaly group, where correct registration and classification is important for surveillance and research. We aimed at reviewing and recoding LRD cases in the MBRN using the same classification system for all years, and evaluate time trends, characteristics and risk factors, 1970–2016.

**Methods:**

After reviewing and recoding LRD cases using International Classification of Diseases (ICD), 10^th^ version, for all years, time trends, association with major anomalies, risk factors and infant outcomes were calculated. Generalized linear models for the binomial family with log link gave relative risks (RR) with 95% confidence intervals (CI). Classification of LRD as suggested by European surveillance of congenital anomalies (EUROCAT) was attempted.

**Results:**

Overall LRD prevalence, 1970–2016, was 4.4 per 10 000, slightly increasing during 1970–1981, followed by relatively stable rates. There were more defects in upper than lower limbs. Defects in hands/fingers were most common, but unspecific descriptions prevented classification of LRD according to EUROCAT. A majority of cases had associated anomalies, the most common being other limb defects, followed by cardiac defects and anomalies in the nervous and digestive systems. From 1999, 26% of LRD cases were terminated, more than 90% of these had associated major anomalies. Stillbirth, neonatal and infant mortality were higher among infants with LRD, also related to associated anomalies. Pre-gestational diabetes was associated with a more than three times increased risk of offspring total LRD, while no association with maternal epilepsy was found. Taking folate/multivitamin supplements before and/or during pregnancy was associated with lower risk of offspring LRD (adjusted RR 0.7; 95% CI 0.6–0.9), while daily smoking did not significantly increase the risk.

**Conclusion:**

The MBRN now has information on LRD coded by ICD-10 from 1970, but information is not specific enough to use other recommended classification systems. Collecting radiographic descriptions and/or more details from hospital records would improve the quality of the registry data. Taking folate supplements before/during pregnancy may reduce the risk of offspring LRD.

## Introduction

In the late 1950’ies, Thalidomide was released as a sedative and antiemetic drug, marketed for treating morning sickness in pregnant women. By 1962, it was withdrawn from use over most of the world after having caused congenital anomalies, especially limb reduction defects (LRD), in more than 10 000 children globally [1]. In the aftermath of this disaster, the Medical Birth Registry of Norway (MBRN) was established in 1967 as the first national, medical birth registry globally, with epidemiological surveillance of congenital anomalies and other adverse perinatal health outcomes as a main aim [2]. With this background in mind, the MBRN should have a special interest in LRD.

From 1967 to 1998, births were notified to the MBRN using a standard paper notification form, where health information, including offspring congenital anomalies, was notified as free text descriptions. Free text was coded at the registry using the International Classification of Diseases (ICD), version 8 (ICD-8), and internal codes if needed. LRD cases were coded by ICD-8 during the years 1967–1987, with codes for reduction defect upper limb, lower limb and unspecified limb, and by internal codes during 1988–1998. The internal codes specified transverse, preaxial, postaxial, and intercalary reduction defects, but unfortunately without separating defects in upper and lower limbs. In 1999, the MBRN switched from ICD-8 to ICD-10, with the British Paediatric Association’s supplement (ICD-10-BPA), for coding congenital anomalies. LRD codes are here much more detailed than in ICD-8.

The previous National Resource Centre for Dysmelia (closed in 2014, activities continued in the National Professional Network for Dysmelia, Norwegian National Advisory Unit on Rare Disorders TRS) had as one of its mandates to build a knowledge base for patients with congenital upper and lower limb anomalies (CULA/CLLA), where LRD constitute an important subgroup. The centre has supported the MBRN in its quality work on LRD, enabling the registry to review all cases registered from 1970 to 1998 and recode these using ICD-10. Since this version of the ICD is more detailed than ICD-8 and defects in upper and lower limbs are separated, the quality of LRD registration could be improved. The overall aim of the collaboration was that the MBRN after the recoding would provide a better basis for research on these rare congenital anomalies.

This paper describes the results of the recoding, and evaluates the recoded data by describing the distribution and time trends for the different reduction defects, their associated anomalies, relations of LRD with mortality and certain risk factors, while comparing the results with the literature.

## Materials and methods

The MBRN is based on mandatory notification of all births in the country, including stillbirths and late fetal deaths from 16 weeks of gestation [2]. From 1999, it also receives mandatory notifications from neonatal intensive care units (NICU) for all infants transferred to such units after birth, as well as notifications of all late (after 12 gestational weeks) terminations of pregnancy due to fetal anomaly (TOPFA). For the TOPFA cases, information to the MBRN is based both on the application for termination, where the gynaecologist describes the anomalies found on antenatal examination (by ultrasound, chromosomal tests or other investigations) as well as on autopsy reports for those TOPFA cases where an autopsy is performed after termination (on average around 50%, somewhat varying between anomaly subgroups). Both these additions (NICU notifications and TOPFA reports) have increased the quality of congenital anomaly registration in the MBRN from 1999. The registry receives information on demographics, maternal chronic diseases, previous pregnancies, diseases and complications during pregnancy, complications and interventions during delivery, and birth outcomes (the child’s vital status, Apgar scores, anthropometric measurements, and neonatal diagnoses, including congenital anomalies). Since 1999, information on mother’s use of folate and multivitamin supplements before and during pregnancy, maternal smoking habits, and gestational age based on ultrasound measurements is also registered, as well as information on mother’s medication use in pregnancy. However, timing of medication use is not specified, and therefore first trimester exposures are not readily defined. Further, the MBRN is routinely linked with the National Registry, which provides all registered individuals their unique national identification numbers and dates of death. The Personal Health Data Filing System Act [3] and the Medical birth registry regulation [4] provide the legal basis for the registry.

**Fig 1:**
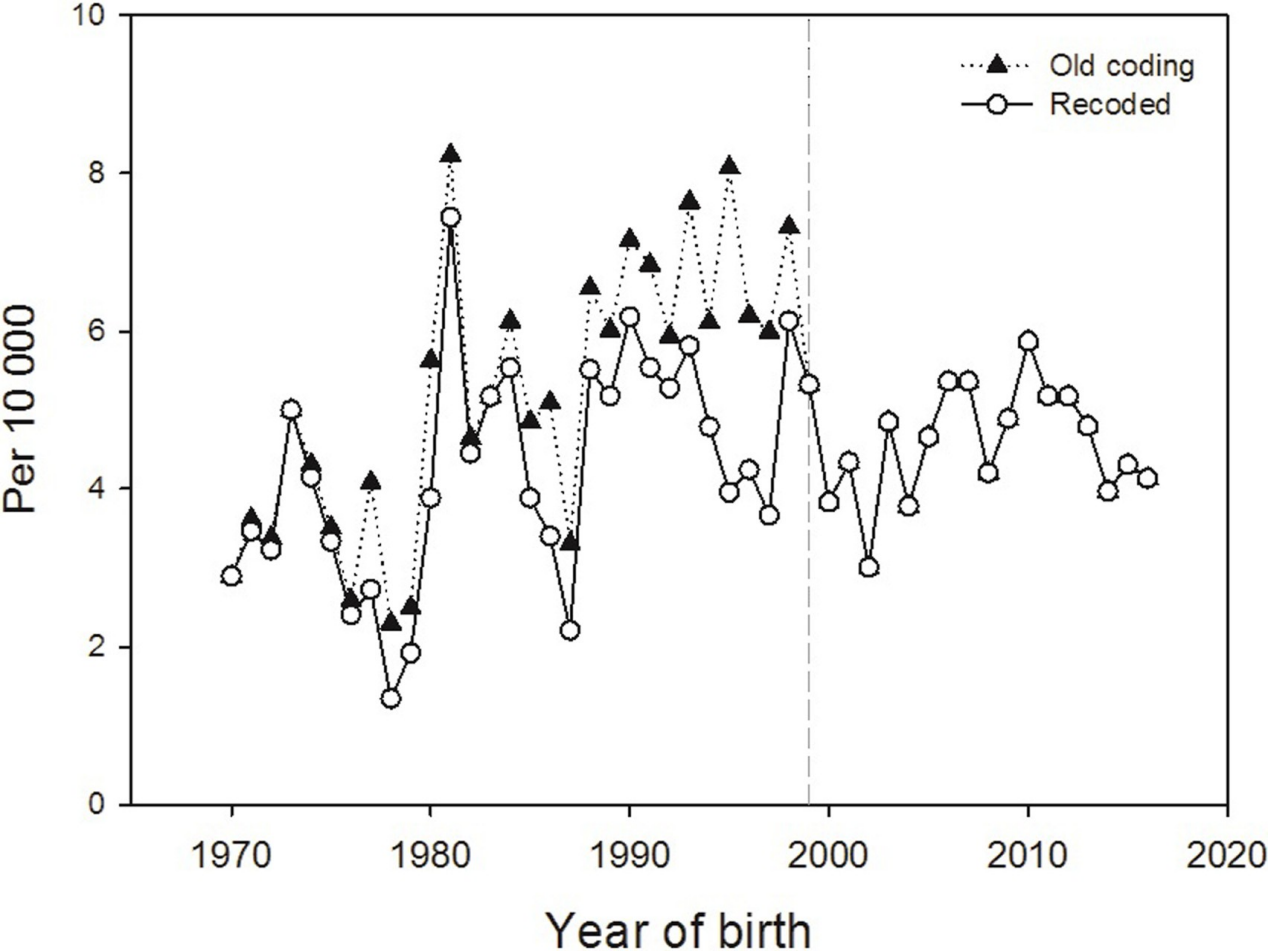
Time trends in overall prevalence (per 10 000) of limb reduction defects, 1970–2016, using original coding (ICD-8 and internal codes during 1970–1998, ICD-10 from 1999) and new coding (ICD-10 for all years). Year 1999 marks changes in the MBRN that have increased ascertainment of congenital anomalies.

LRD prevalence per 10 000 births in the MBRN based on the original registered codes (ICD-8 and internal codes) showed an evident increase over time, especially for boys, until 1998, and then a marked drop (overall trends shown in Fig 1, “old codes”). The drop in 1999 coincides with the mentioned additions in the MBRN (NICU notifications and TOPFA reports), which have generally led to a better ascertainment of congenital anomalies and therefore an increase in prevalence for most anomalies from 1999. The time trends in “old codes” shown in Fig 1 showed the opposite pattern and underlined the need to re-evaluate the coding of LRD in the MBRN for the years before 1999.

All paper birth notifications from 1970 to 1998 where infants were registered with LRD were collected from storage, reviewed and recoded to ICD-10 by two medical doctors at the MBRN (KK and TN) on the basis of free text descriptions on the forms. When descriptions were unspecific or unclear, the cases were discussed with a paediatric orthopedic surgeon (TSK or SS). Results of the recoded data were then analysed using anonymous data files. The legal basis for this quality assurance project was the Medical birth registry regulation (4), which states that the Norwegian Institute of Public Health (where the registry is located) is responsible for ensuring that the data collected and handled at the MBRN are correct, relevant and needed to achieve the aims of the registry. Such quality assurance projects, carried out within the registry, are exempt from ethical evaluation.

We aimed at using the guide “Description and classification of congenital limb defects” by Stoll et al [5], built on a previous suggested classification [6], and used by the European Surveillance of Congenital Anomalies (EUROCAT), a European network of population based congenital anomaly registries where the MBRN is a member. In this guide, LRDs are grouped as shown in Table 1, where we also include our suggestion of possible ICD-10 codes. For classifying associated anomalies, we used EUROCAT definitions to specify subgroups of major anomalies, mainly organ-specific groups [7].

**Table 1:** Guide to description and classification of congenital limb defects [5], with our suggestion of possible ICD-10 codes.

Group	Subgroups	Suggested ICD-10 codes
Terminal Transverse Defects: Absence of distal structure of the limb with proximal structures more or less normal		
	Amelia–total absence of the extremities	Q710, Q720, Q730
	Hemimelia–total absence of forearm and hand or of foreleg and foot	Q712, Q722
	Acheiria–absence of hand	Q713
	Apodia–absence of foot	Q723
	Adactyly–absence of digits	Q713, Q723
	Ectrodactyly–total or partial absence of phalanx	Q713, Q723
Proximal-intercalary Defect: Absence or severe hypoplasia of proximal-intercalary part of the limb when the distal structures (ie. the digits), whether normal or malformed, are present:		
	Absence of humerus and/or radius and ulna (with hand normal or near normal)	Q711
	Absence of femur and/or tibia and fibula (with foot normal or near normal).	Q721, Q724
Longitudinal Defects: Absence or severe hypoplasia of lateral part of the limb:		
	Preaxial (Radial-tibial)–absence or severe hypoplasia of preaxial structures of the limb (thumb, first metacarpal, radius: hallux, first metacarpal, tibia).	Q714, Q725
	Postaxial (Ulna-fibula)–absence or severe hypoplasia of postaxial structures of the limb (little finger, 5th metacarpal, ulna; 5th toe, 5th metatarsal, fibula).	Q715, Q726
Split Hand-foot: Absence of central digits with or without absence of central metacarpal/ metatarsal bones usually associated with syndactyly of other digits.		
	Typical split hand/foot: cone-shaped cleft tapering proximally and dividing the hand into 2 parts, which can be opposed like lobster-claws. In the mildest forms the 3rd digit (middle finger or 3rd tow) is absent but the corresponding metacarpal/ metatarsal bone is almost normal.	Q716, Q727
	Monodactyly of the hand is characterised by deficiency of the central and radial digits, such that there is no cleft formation and only one digit is present (usually the 5th).	Q716, Q727
Multiple Types of Reduction Defects: Infants with more than one type of reduction according to the classification given above.		

After recoding, we evaluated results by analysing time trends of LRD prevalence per 10 000 births from 1970–2016, overall and for upper, lower and unspecified limb, and we calculated the frequency of specific diagnoses. We also described associated major congenital anomalies during 1970–1998 and 1999–2016, overall and for TOPFA cases, stillbirths and neonatal deaths from 1999. We evaluated some potential risk factors for offspring LRD, such as maternal age and parity as well as maternal pre-gestational diabetes, maternal epilepsy and, from 1999, smoking habits and use of folate/multivitamin supplements. Finally, stillbirth rates (per 1000 births), neonatal mortality and infant mortality (per 1000 live births) among LRD cases with and without associated anomalies were compared with rates in infants without LRD. We also compared low birth weight (LBW, birth weight < 2500 grams), preterm delivery (< 37 completed weeks of gestation) and male sex among cases with and without LRD.

### Statistics

We used frequency tables and generalized linear models for the binomial family with log link to estimate crude and adjusted relative risks (RR) with 95% confidence intervals (CI). Time trends were judged by evaluating linear trends in the multivariate models. The following variables were evaluated as possible confounding factors (varying depending on the specific analysis, see table footnotes): year of birth (four-year groups), maternal age (<20, 20–24, 25–29, 30–34, 35–39, 40+ years), maternal pre-gestational diabetes (yes/no), sex, and for data from 1999: smoking habits (daily smoking yes/no), and use of folate/multivitamin supplements (yes/no). There were 19% missing in the smoking variable, and 12% missing in the folate/multivitamin variable, however, these were not missing at random as the TOPFAs had 91% missing for smoking and 45% missing for folate/multivitamins. Information on maternal chronic diseases was also missing for most TOPFAs. When analysing the associations of maternal diabetes, epilepsy, smoking and folate/multivitamin supplements with LRD, we therefore excluded TOPFAs. We then used multiple imputation with chained equations and 20 imputations to impute missing data in smoking and folate/multivitamins. Variables used in the imputation were the model variables and mother’s marital status, birth year, mother’s birth year, birth weight, gestational age, perinatal death, preeclampsia, use of assisted reproduction technology, all significantly associated with smoking and folate/multivitamin supplements. Two-sided tests with a significance threshold of *P*<0.05 were used in all analyses. We used SPSS version 25 (IBM Statistical Package for the Social Sciences, SPSS Inc, Chicago, IL, USA) and STATA intercooled v.14 (StataCorp LP 2015. Stata Statistical Software: Release 14) for analyses.

## Results

### Prevalence and time trends

The free text descriptions of congenital anomalies on the birth notification forms, 1970–1998, varied in detail and could be unspecific. For example, the free text “lacks one finger” could, depending on which finger, be recoded using Q713 (Congenital absence of hand and finger(s)), but also Q716 (Lobster-claw hand) if the missing finger was the third, Q714 (longitudinal reduction defect, radius) or Q715 (longitudinal reduction defect, ulna) if the first or fifth. Several cases had been coded with LRD codes, but where free text descriptions were not specific for a reduction defect. For most of these cases, we used other codes, e.g. “Deformed hand” which had been coded by ICD-8 code 7552 (reduction defect upper limb), was recoded using Q681 (Congenital deformity of hand). These situations were discussed with an orthopedic surgeon.

A total of 1 369 cases were registered with LRD in the MBRN from 1970 to 2016 before recoding. After recoding cases in 1970–1998, this number was reduced to 1 206. In the same period, a total of 2 745 119 births were registered in the MBRN, giving an LRD prevalence of 5.0 per 10 000 before recoding, and 4.4 per 10 000 afterwards. For 1970–1998, a total of 869 cases were registered with LRD before recoding (5.2 per 10 000), which was reduced to 706 (4.2 per 10 000) after recoding. During 1999–2016, the total number of registered LRD cases was 500 with prevalence 4.6 per 10 000, and the recoded data thus showed the expected increase in prevalence for the years 1999–2016 relative the years before.

In the last time period (1999–2016), 129 of the 500 LRD cases (25.8%) were TOPFAs, and 140 of 352 live born infants with LRD (39.8%) were transferred to a NICU after birth.

Time trends in prevalence of LRD per 10 000 births as registered in the MBRN before and after recoding are shown in Fig 1. After recoding, the increase in prevalence from 1970 to 1998 with the sudden decrease thereafter was no longer as evident as before. Over the full time period (1970–2016), there was a slight increase in recoded LRD prevalence (test for trend, *P*<0.01, Wald test), seemingly due to a statistically significant increase in the first decade with a marked high prevalence in 1981 (7.4 per 10 000). During 1982–2016, there was no statistically significant linear trend over time, (test for trend, *P* = 0.9, Wald test).

**Fig 2:**
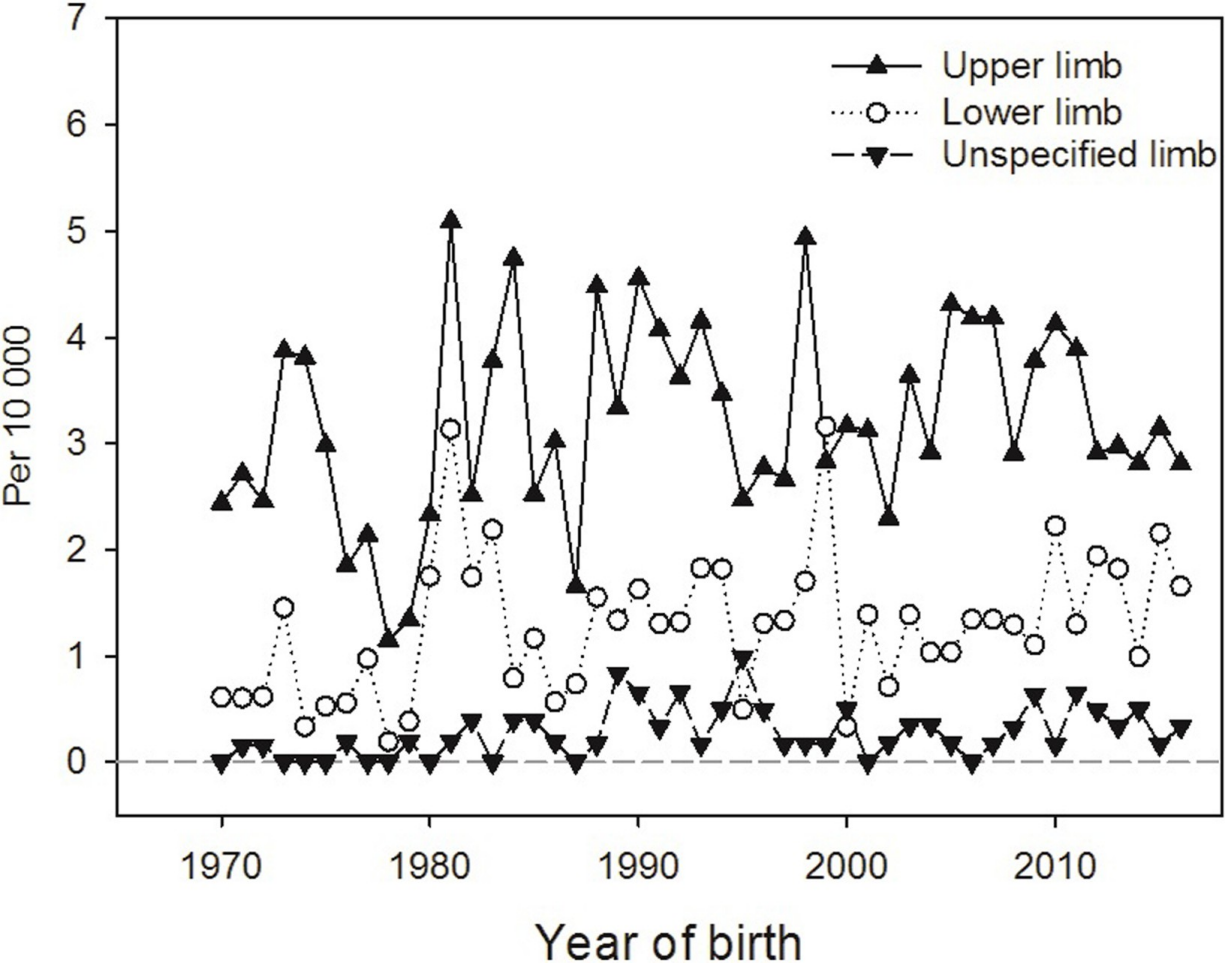
Prevalence (per 10 000) of limb reduction defects in upper, lower and unspecified limb, 1970–2016.

Among all 1 206 recoded LRD cases, 783 (64.9%) had defects registered in the upper limbs only, 249 (20.6%) in the lower limbs only and 102 (8.5%) in both upper and lower limbs. In 72 cases (6.0%), the affected limbs were not specified as being upper or lower. Fig 2 shows prevalence, 1970–2016, for defects in upper, lower and unspecified limb (those with both limbs affected shown twice). The prevalence of reduction defects in the upper limbs was slightly more than twice that in the lower limbs during the entire period, with overall prevalences being 3.2 and 1.3 per 10 000, respectively. For 86% of all cases, only one reduction defect was registered. The most common defect was Q713: “Congenital absence of hand and finger(s)”, registered in 392 of the 1 206 cases (32.5%). The corresponding code in the lower limb, Q723: “Congenital absence of foot and toe(s)”, was registered in 135 individuals (11.2%). “Congenital absence of both forearm and hand” (Q712) and “Longitudinal reduction defect of radius” (Q714) were both also relatively frequent, and registered in 10.5% and 11.8%, respectively, see S1 Table.

We had aimed at classifying the reduction defects using the classification system shown in Table 1, but due to unspecific descriptions of defects in the hands and feet where the exact finger(s) and toe(s) affected were often not specified, this proved impossible.

### Associated anomalies

**Fig 3:**
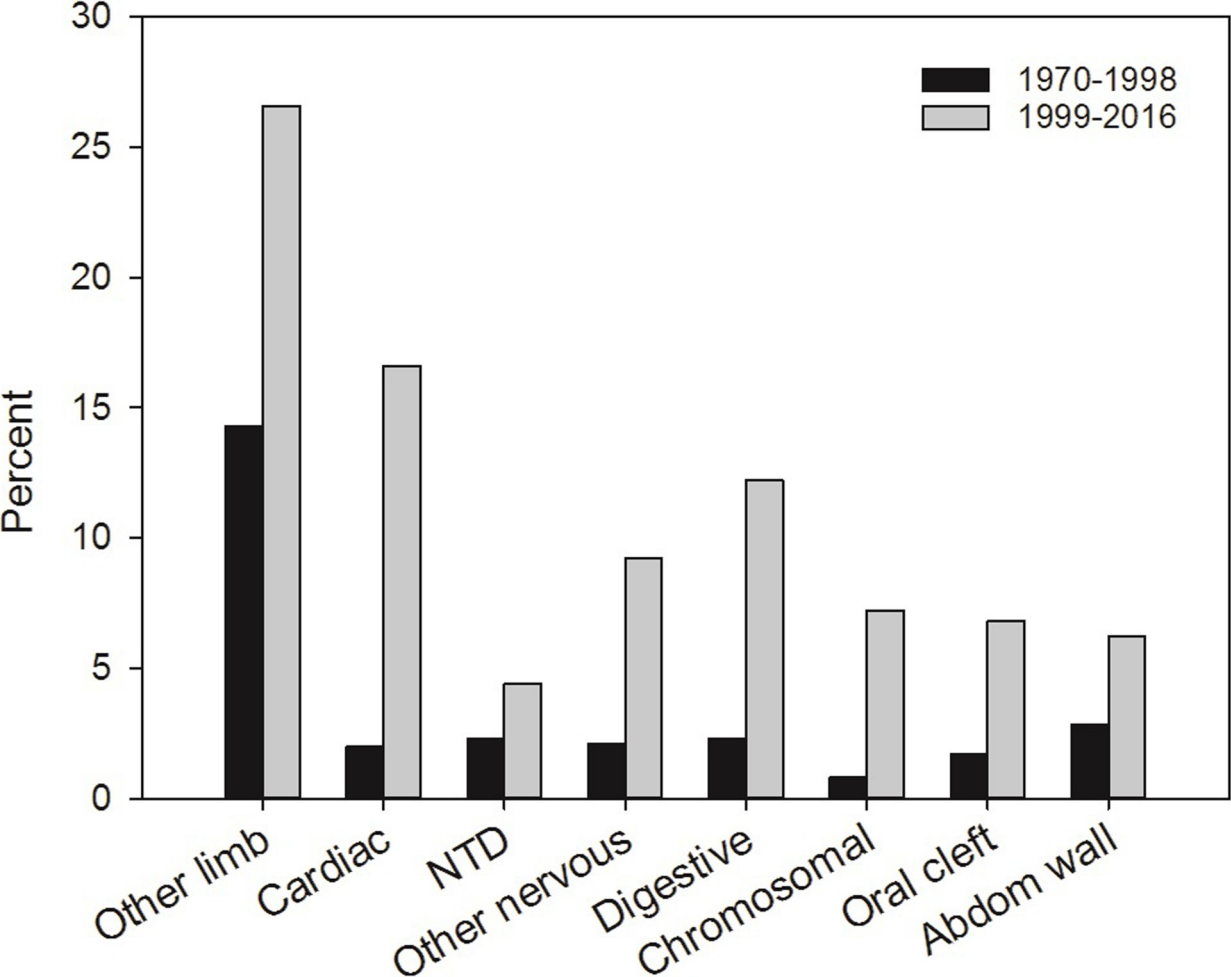
Distribution of associated major congenital anomalies (%) among limb reduction defect cases in 1970–1998 and 1999–2016.

For LRD cases with associated major anomalies, there was a marked difference between the years before and after 1999: During 1970–1998, associated major anomalies were registered in 26.3%, while this increased to 53.2% in the following years (1999–2016). Fig 3 shows the distribution of associated anomalies (percent) for LRD cases in 1970–1998 and 1999–2016. In both periods, other limb anomalies dominated: 14.3% in the first time period and 26.6% in the last. Among these, syndactyly was the most frequent, 39% and 41% of the other limb anomalies in the first and last period, respectively, followed by club foot (pes equinovarus) with around 25% of other limb anomalies in both periods, while polydactyly was found in 5.0% (1970–1998) and 11.3% (1999–2016). From 1999, cardiac defects followed other limb defects in frequency (16.6% of the 500 LRD cases), then nervous system anomalies (13.6%) and anomalies of the digestive system (12.2%). Chromosomal syndromes were registered in 36 of the 500 LRD cases (7.2%) in the last time period, where 24 of 36 were trisomy 18. Before 1999, all other anomaly groups except other limb defects were rare, with nervous system anomalies being registered in 4.4%, cardiac defects in 2% and chromosomal syndromes in 0.8% of the LRD cases.

Among 207 cases with associated major anomalies outside limbs in 1999–2016, approximately one third (N = 71, 34%, or 14% of all 500 cases) were registered with acknowledged associations or syndromes (VACTERL association, sirenomelia, amniotic bands, limb-body-wall complex and teratogenic, genetic or chromosomal syndromes). The remaining 136 (27% of all 500 cases) were classified as non-syndromic multiple major anomalies. Among the 429 LRD cases without acknowledged associations or syndromes, the largest group of associated anomalies was cardiac defects (N = 53, 12.4%), followed by anomalies in the digestive and in the nervous system (both N = 40, 9.3%), and oral clefts (N = 19, 4.4%). Among the 40 cases with nervous system anomalies, 14 had neural tube defects.

### Maternal factors

Table 2 shows the distribution of selected maternal factors in the total population and among cases with LRD, all cases, after excluding genetic and chromosomal syndromes, and finally in isolated LRD using data from 1999 (N = 293 overall, 281 excluding TOPFA) due to better registration of associated anomalies after 1998. Prevalence of LRD in strata of maternal factors, with corresponding RRs are also shown. TOPFA cases were excluded except when analysing maternal age and parity, since TOPFA reports have lacked information on maternal factors other than age and parity until recent years. There were significantly more primiparous women among mothers of affected infants, and both being young (<20 years: adjusted RR (adjRR) 1.3 (95% CI 1.01–1.71) and 20–24 years: adjRR 1.2; 1.1–1.4) and old (> = 40 years: adjRR 1.6; 1.2–2.3) when giving birth was associated with increased risk of offspring total LRD. The association with high maternal age was, however, not statistically significant for non-syndromic or isolated LRD. The risk of offspring total LRD was more than three times higher in women with pre-gestational diabetes than in those without (adjRR 3.4; 2.0–5.7), and more than doubled for isolated LRD (adjRR 2.6; 1.1–6.3). There was no significant association between maternal epilepsy and offspring LRD, although only four mothers of isolated LRD cases had epilepsy. For infants born after 1998, daily smoking was at most weakly associated with offspring total LRD (adjRR 1.2; 0.9–1.6), and no relation was found with isolated LRD (adjRR 1.0; 0.7–1.5). Use of folate/multivitamin supplements before and/or during pregnancy was, on the other hand, associated with a 30% lower risk of offspring LRD compared to non-users, both for total (adjRR 0.7; 0.6–0.9) and isolated (adjRR 0.7; 0.6–0.98) LRD.

**Table 2:** Distribution of maternal factors in the total population and among limb reduction defects (LRD) cases, along with LRD prevalence by each factor. Pregnancy terminations due to congenital anomalies are excluded except when analysing parity and maternal age.

Factor	Total population	Total LRD N (per 10 000)	Total LRD Crude RR (95% CI)	Total LRD Adj RR^a^ (95% CI)	Non-syndromic LimbRedDef Adj RR^a^^,^^b^ (95% CI)	Isolated LRD^c^ Adj RR^a^ (95% CI)
**Parity**						
0	1,147,173	612 (5.3)	1.0 (ref)	1.0 (ref)	1.0 (ref)	1.0 (ref)
≥ 1	1,597,939	594 (3.7)	0.7 (0.6–0.8)	0.7 (0.6–0.8)	0.7 (0.6–0.8)	0.7 (0.6–0.9)
**Maternal age (years)**						
< 20	131,431	65 (4.9)	1.2 (0.9–1.6)	1.3 (1.0–1.71)	1.2 (0.9–1.6)	0.8 (0.3–2.1)
20–24	630,424	309 (4.9)	1.2 (1.0–1.4)	1.2 (1.1–1.4)	1.2 (1.1–1.4)	1.4 (1.0–1.9)
25–29	940,827	389 (4.1)	1.0 (ref)	1.0 (ref)	1.0 (ref)	1.0 (ref)
30–34	705,209	285 (4.0)	1.0 (0.8–1.1)	0.9 (0.8–1.1)	0.9 (0.8–1.1)	0.9 (0.7–1.3)
35–39	284,459	121 (4.3)	1.0 (0.8–1.3)	1.0 (0.8–1.2)	0.9 (0.7–1.1)	1.0 (0.7–1.4)
40+	52,759	37 (7.0)	1.7 (1.2–2.4)	1.6 (1.2–2.3)	1.4 (0.9–2.0)	1.2 (0.6–2.3)
**Maternal pre-gestational diabetes**^d^						
Yes	11,698	15 (12.8)	3.3 (2.0–5.5)	3.4 (2.0–5.7)	3.5 (2.1–5.8)	2.6 (1.1–6.3)
No	2,729,202	1,062 (3.9)	1.0 (ref)	1.0 (ref)	1.0 (ref)	1.0 (ref)
**Maternal epilepsy**						
Yes	16,393	8 (4.9)	1.2 (0.6–2.5)	1.2 (0.6–2.5)	1.3 (0.6–2.5)	2.1 (0.8–5.5)
No	2,724,507	1,069 (3.9)	1.0 (ref)	1.0 (ref)	1.0 (ref)	1.0 (ref)
**Maternal daily smoking**^e^						
				1.2 (0.9–1.6)	1.2 (0.8–1.6)	1.1 (0.7–1.5)
Yes	134,351	56 (4.2)	1.3 (0.9–1.7)	1.2 (0.9–1.6)^f^	1.2 (0.9–1.6)^f^	1.0 (0.7–1.5)^f^
No	740,154	246 (3.3)	1.0 (ref)	1.0 (ref)	1.0 (ref)	1.0 (ref)
**Use of folate/multivitamin supplements**^e^						
				0.8 (0.6–1.02)	0.8 (0.6–1.01)	0.8 (0.6–1.07)
Yes	661,066	195 (2.9)	0.7 (0.6–0.9)	0.7 (0.6–0.9)^f^	0.7 (0.6–0.9)^f^	0.7 (0.6–0.98)^f^
No	287,249	122 (4.2)	1.0 (ref)	1.0 (ref)	1.0 (ref)	1.0 (ref)

Abbreviations: Limb reduction defects (LRD, LimbRedDef); Relative risk (RR); Confidence intervals (CI); Reference category (ref)

^a^ For parity and maternal age, RRs were adjusted for year of birth (4-year groups). For other factors, adjustments also included maternal age as categorized in the table, and for smoking and folate/multivitamin supplements, both factors were included, in addition to maternal age and diabetes;

^b^ Excluding cases with chromosomal and other syndromes;

^c^ Isolated LRD (N = 293 with and 281 without TOPFA), using data from 1999 due to better registration of associated anomalies after 1998.

^d^ Pre-gestational diabetes type 1, 2 and unspecified;

^e^ Data from 1999, missing smoking information 19%, missing folate information 12%;

^f^ Results when using multiple imputation to handle missing data in smoking and folate/multivitamins

Multiple congenital anomalies without known underlying causes may be a marker of teratogenic exposures [8, 9]. We therefore repeated analyses for maternal pre-gestational diabetes, daily smoking, and use of folate/multivitamin supplements looking at non-syndromic cases of LRD associated with other major anomalies (“LRD-multiples”). After excluding TOPFAs, there were only 72 cases of LRD-multiples, and for this group the adjusted RR associated with pre-gestational diabetes was 6.3 (2.0–20.1), for daily-smokers relative non-smokers 1.4 (0.7–2.6) and for women using folate/multivitamins supplements relative non-users 0.6 (0.4–1.1).

### Pregnancy termination, mortality and offspring characteristics

During 1999–2016, a total of 129 of 500 LRD cases were TOPFA (25.8%). Among these, associated anomalies were common, although 12 (9.3%) were registered with isolated LRD. On the other hand, a majority (77.5%) of the TOPFA cases had more than one associated anomaly, and in 31 cases (24%), major anomalies were registered in three or more additional subgroups. Other limb defects dominated also among TOPFA cases and were found in 40.3%, after which followed urinary defects (36.4%), cardiac defects (34.1%) and nervous system defects (33.3%), while chromosomal syndromes were found in 28 cases (21.7%). Of the total 129 TOPFA cases, 100 (77.5%) had an autopsy after termination, while five of the 12 TOPFA cases with isolated LRD (41.7%) had an autopsy.

After excluding TOPFA cases from 1999–2016, we analysed mortality among the remaining 1077 LRD cases for all years, 1970–2016 (Table 3, A) and, after also excluding associated major non-limb anomalies, for the remaining 281 cases born 1999–2016 (B). The last period was chosen since associated major anomalies were better registered from 1999. Overall, a total of 84% LRD cases were alive in 2016, based on updated data from the National registry, stillbirth was registered in 7.9% of cases, and among livebirths, 4.2% died during the first month (neonatal deaths) and 5.5% during the full first year (infant deaths). Thus, the risk of stillbirth was 7.4 times increased, neonatal mortality around 10 times increased and infant mortality 8.6 times increased compared to infants without LRD. However, after excluding associated major anomalies in other organs and looking only at infants born in 1999–2016, LRD did not seem to be associated with an increased mortality risk, although number of deaths were very few and confidence intervals were wide.

**Table 3:** A: Mortality, low birth weight, preterm birth, and male sex among 1077 infants with limb reduction defects (LRD) relative infants without LRD during 1970–2016, terminations 1999–2016 excluded. B: Same outcomes among 281 infants with LRD relative infants without, 1999–2016, excluding terminations and LRD associated with major anomalies in other organs.

**A**	**Total LRD (1970–2016)**		**Crude RR (95% CI)**	**Adjusted RR**^a^ **(95% CI)**
**Outcomes**	**No (reference)**	**Yes**		
Mortality				
Stillbirth (N, per 1000)	26,187 (9.6)	85 (78.9)	8.3 (6.7–10.1)	7.4 (6.0–9.3)
Neonatal death^b^ (N, per1000)	10,909 (4.0)	42 (42.3)	10.5 (7.8–14.2)	10.1 (7.6–13.6)
Infant death (N, per 1000)^c^	16,537 (6.1)	55 (55.4)	9.1 (7.0–11.8)	8.6 (6.7–11.1)
Birth weight				
<2500 grams (N,%)	142,262 (5.2)	224 (20.8)	4.0 (3.6–4.5)	3.9 (3.4–4.3)
Gestational age				
< 37 weeks (N, %)	179,396 (6.4)	201 (18.7)	2.9 (2.6–3.3)	2.7 (2.4–3.1)
Sex^d^				
Male (N, %)	1,407,721 (51.4)	609 (57.1)	1.11 (1.05–1.17)	
**B**	**LRD without associated non-limb anomalies (1999–2016)**		**Crude RR (95% CI)**	**Adjusted RR** ^a^^,^^e^ **(95% CI)**
**Outcomes**	**No**	**Yes**		
Mortality				
Stillbirth (N, per 1000)	8,000 (7.4)	5 (17.8)	2.4 (1.0–5.7)	2.0 (0.8–5.4)
Neonatal death (N, per 1000)^b^	2,262 (2.1)	0	NA	NA
Infant death (N, per 1000)^c^	3,193 (3.0)	1 (3.6)	1.2 (0.2–8.6)	1.1 (0.2–8.0)
Birth weight				
<2500 grams (N, %)	57,344 (5.3)	36 (12.8)	2.4 (1.8–3.3)	2.3 (1.7–3.1)
Gestational age				
< 37 weeks (N, %)	76,385 (7.1)	39 (13.9)	2.0 (1.5–2.6)	1.8 (1.4–2.5)
Sex				
Male (N, %)	552,764 (51.4)	158 (56.4)	1.10 (0.99–1.22)	

Abbreviations: Limb reduction defects (LRD); Relative risk (RR); Confidence intervals (CI); Not applicable (NA)

^a^ Adjusted for time period (4-year categories), sex, maternal age (as categorized in Table 2) and maternal pre-gestational diabetes (yes/no);

^b^ First month of life;

^c^ First full year of life;

^d^ One stillbirth had unknown/unspecified sex;

^e^ Additional adjustment for maternal smoking (yes/no) and use of folate supplements (yes/no)

Among a total of 85 stillbirths with LRD, 59 (69.4%) were registered with associated anomalies; 45 of 66 (68%) in 1970–1998 and 14 of 19 (74%) in 1999–2016. Nervous system anomalies, other limb anomalies and abdominal wall defects were the most common associated anomaly groups in stillbirths in both periods. Among neonatal deaths, 39 of 42 (93%) were registered with associated anomalies (32 of 35 (91.4%) in 1970–1998 and all seven in 1999–2016). Here the most common associated anomalies in both periods were nervous system defects and cardiac defects.

The table also shows LBW, preterm delivery and male sex in the total population and in the two groups of LRD infants. LBW and preterm delivery was more common among affected infants, also among the cases without associated major anomalies from the last time period. As expected, there were more male cases, however, the male excess was slightly smaller among cases without associated major anomalies in the last time period.

## Discussion

This paper describes the results from a review and recoding of LRD cases in the MBRN, 1970–1998, and analyses done to evaluate the results of the recoding. The overall LRD prevalence, 1970–2016, was 4.4 per 10 000, with a slight increase in prevalence during the first decade, and relatively stable rates from 1982. There were more defects in upper than lower limbs, with defects in the hands and fingers being the most common. A large proportion of cases had associated anomalies, the most common being other limb defects. Associated anomalies were far better registered in the MBRN from 1999, and in this period, around one third of those with associated non-limb anomalies had acknowledged associations/syndromes. Among the non-syndromic cases, cardiac defects were the most common associated major anomalies, followed by anomalies in the nervous and digestive systems. From 1999, nearly 26% of limb reduction cases were terminated, however, more than 90% of these had associated major anomalies, with urinary defects, cardiac defects and nervous system defects being most frequent. Mortality was significantly higher among infants with LRD, but again, most of the increased mortality was related to associated major anomalies. Finally, we found that taking folate/multivitamin supplements before and/or during pregnancy was associated with lower risk of offspring LRD among children being born, while maternal daily smoking did not significantly increase the risk.

The overall prevalence of LRD in our study (4.4 per 10 000 in 1970–2016 and 4.6 per 10 000 in 1999–2016) was in agreement with a recent study by the EUROCAT association, describing European trends in congenital anomalies, 1980–2012 [10]. The overall prevalence for LRD in that study was 4.5 per 10 000 during 2003–2012, however, with an overall decreasing time trend during these years, all registries merged. Norway, being part of EUROCAT, was one of the registries where no significant decreasing time trend was found, along with registries in Ireland, Zagreb, Antwerp, Tuscany and Hungary. South Portugal, Basque country and Paris had increasing prevalence trends during the same years, however, the overall conclusion was that the decreasing pan-European trend was likely a true trend, but without known causes [10]. The same paper described a clearly higher rate of LRD in many of the European registries in 1989/90, likely associated with early chorion villus sampling [11]. In our data, the highest prevalence was in 1981, without a known cause.

In a Canadian study reviewing data from the Alberta Congenital Anomalies Surveillance System (ACASS), Bedard et al reported an average prevalence rate of congenital LRD at 5.6 per 10 000 births, 1980–2012 [12]. They also reported, in agreement with our findings, higher proportions of upper than lower limb defects. Their 63.9% in upper limbs, 25.3% in lower limbs and 10.8% in both were close to the corresponding 64.9%, 20.6% and 8.5% in our data. The authors classified the LRD cases using a classification system similar to that shown in Table 1. We had aimed at classifying our LRD cases according to this system, but were unable to do so because the free text descriptions were often not specific enough, especially concerning defects in hands and feet. Since causes of LRD may vary between specific groups of defects, correct classification is important, and additional information including results of radiographs would be desirable when the MBRN receives future notifications of LRD. Bedard et al rated the quality of the LRD data in their study as high (radiograph or necropsy with detailed clinical description), medium (no radiograph, but detailed clinical description) and low (general description only) [12]. If applied to the MBRN data, a high proportion of diagnoses would be rated as having low quality, especially during 1970–1998. From 1999, autopsy reports are routinely received for a large proportion of stillbirths and TOPFAs, but radiographs or detailed descriptions of affected bones in live born infants are usually not.

In a registry-based study from Finland, where data from the Finnish Register of Congenital Malformations (FRCM) was used to describe CULA, the national incidence was 5.56 per 10 000 births, and 5.25 per 10 000 live births [13]. In a detailed and more clinical, population based study from Stockholm, the incidence of CULA was as high as 21.5 per 10 000 live births [14]. These rates were significantly higher than our 3.2 per 10 000 for upper LRD, however, not only reduction defects were included in the Stockholm study: “Trigger digit or thumb” in the “Failure of differentiation” group and polydactyly were the most frequent there, but also camptodactyly and syndactyly were common. Our study, focusing on reduction defects, only included other limb defects when associated with LRD. The “Failure of formation” group in the Stockholm study corresponds to our upper limb reduction defects, and the prevalence for this group was 3.9 per 10 000 [14], which is close to our result. However, some underreporting of LRD to the MBRN is likely, especially for milder defects like thumb hypoplasia (mild radial longitudinal deficiency) and mild (sym-)brachydactylia.

Many previous studies have described associated anomalies in patients with LRD, but with a large range [12, 13, 15–19]. In our study, associated anomalies were poorly registered during 1970–1998. The changes in 1999 increased the quality of congenital anomaly registration in the MBRN, and our results concerning associated anomalies are most valid for the years 1999–2016. Evans et al described associated anomalies in a study from Hungary in 1994, where structural anomalies in other organ systems than musculoskeletal were found in 33.1% [18]. In this study, a great majority of the cases with associated anomalies had known causes. Bedard et al found associated anomalies in 56.4% of limb reduction defect cases, after excluding a number of other musculoskeletal anomalies and deformities including isolated syndactyly and polydactyly [12]. When also excluding associations that were due to acknowledged causes (well known associations, teratogenic, genetic and chromosomal syndromes), the proportion of multiple congenital major anomalies was 21.9%. Stoll et al found non-syndromic multiple major anomalies in 28.8% of patients with LRD [17]. Both of these studies are in the range of our 27% non-syndromic multiple major associated anomalies.

We found that 7.2% of LRD cases 1999–2016 were associated with chromosomal syndromes, compared to 6.3% in the study by Stoll et al [17], and 5.4% in the study by Bedard et al [12]. The slightly higher proportion in our study could be related to our data being more recent with births up to 2016, since maternal age has been increasing steadily since the 1970’ies and is an important cause of chromosomal abnormalities. In agreement with other studies, trisomy 18 was the most common associated chromosomal abnormality in our data. Also in agreement with Stoll et al, we found that among the non-syndromic congenital anomalies associated with LRD, cardiac defects were the most frequent with 12.4% (among 429 non-syndromic cases) in our study compared to 11.4% in the study by Stoll et al [17]. The study by Bedard et al from 2018, describing LRD cases with at least one associated major anomaly, also found gastrointestinal anomalies to be common, in agreement with our results [19]. Both studies by Bedard et al analysed associated anomalies by subgroups of LRD [12, 19], which we unfortunately could not do in a similar manner due to the mentioned problems of classifying defects due to unspecific information concerning which fingers/toes were affected.

LRDs are known to be associated with teratogenic exposures during pregnancy, such as maternal medication, where Thalidomide is the most well known. Other examples include antiepileptic/anticonvulsant drugs like phenytoin and valproic acid [20–22]. Maternal diabetes is associated with several congenital anomalies in the offspring, including LRD [23–25], likely due to hyperglycaemic episodes. A study from the EUROCAT network did not find an association between maternal diabetes and LRD, but this study did not have data on non-malformed infants [26]. We found offspring total LRD to be more than three times more frequent among mothers with than without pre-gestational diabetes, and isolated LRD more than doubled, thus supporting most previous reports. Maternal epilepsy, on the other hand, was not associated with offspring LRD in our data, although the point estimate for isolated LRD in the last time period was compatible with a possible association. However, with only four exposed LRD infants (where mothers had epilepsy), we had inadequate power to conclude regarding a possible association between maternal epilepsy and isolated LRD. In their paper from 2001, Holmes et al concluded that an association between maternal epilepsy and offspring LRD is likely due to teratogenic effects from antiepileptic medication taken in pregnancy rather than the epilepsy itself [21]. Mothers with epilepsy registered in the MBRN include also mothers that have had epilepsy previously but did not need medication during pregnancy, which may explain our findings.

We did not find a statistically significant association between maternal smoking and offspring LRD. This is in contrast to several previous studies [27–30], and summed up by a systematic review and meta-analyses based on 173 687 malformed cases and 11.7 million controls where the aim was to evaluate the relation between maternal smoking and several birth defects [31]. This review found that maternal smoking was related to offspring LRD with a pooled odds ratio of 1.26 (95% CI 1.15–1.39). We found a point estimate close to this for total LRD, however, confidence intervals included 1.0 (adjRR 1.2; 95% CI 0.9–1.6). We may have lacked power, since we could only include births from 1999 (when MBRN included smoking data), and we had to exclude TOPFA cases where more than 90% lacked information on maternal smoking. For isolated LRD, however, there was no association.

We found a 30% decreased risk of offspring total and isolated LRD associated with mothers taking folate/ multivitamin supplements before and/or during pregnancy (adjRR 0.7; 95% CI 0.6–0.9 and 0.6–0.98, respectively). This is close to what Shaw et al found in a small case-control study from 1995, where the odds ratio was 0.64 (0.41–1.0) [32]. Canfield et al studied whether grain fortification with folic acid had affected rates of several birth defects, including LRD, using data from 23 state member programs in the National Birth Defects Prevention Network (NBDPN) to analyse prevalence rates before and after grain fortification was implemented [33]. They found a small, but statistically significant reduced prevalence of upper LRD when comparing post- versus pre-fortification rates. Other studies that have found preventive effects of folate or multivitamin supplements containing folate include the Hungarian double blind placebo controlled trial [34], an American case control study on multivitamin supplementation and risk of several birth defects where 31 LRD cases were included [35], and a study using data from the population based Atlanta Birth defects Case Control study where effects were found mainly for transverse limb defects [32]. A systematic review and meta analysis from 2017 summed up studies on multivitamin use and adverse birth outcomes and found a summary relative risk for limb deficiencies at 0.68 (95% CI, 0.52–0.89) [36], very close to our findings. Other indications of folate playing a causal role in relation to LRD, are studies looking at folate pathway polymorphisms, such as the study by Cleves et al [37]. However, some evidence is less supportive regarding the preventive effect of folate on LRD. A case control study from Australia on primary prevention of non-neural birth defects where 26 LRD cases were included, concluded that neither folic acid supplements nor dietary folate intake was significantly associated with the risk of offspring LRD [38], however, confidence intervals were wide. Further, a study using data from the NBDPN found no association between folate supplements and LRD [39]. This study was, however, carried out after fortification of flour with folic acid was implemented, and did find that among women not using supplements, a diet poor of folate or vitamin B6 was associated with an increased odds of transverse limb defects. In Norway, fortification of flour with folic acid is not implemented, but there are official recommendations that all women planning pregnancy should take a daily supplement of 0.4 mg folic acid from one month before until 12 weeks after conception in order to reduce the risk of offspring NTD. Our results indicate that this recommendation may also be important for the risk of offspring LRD, especially in a situation without fortification of the food supply.

When comparing non-syndromic LRD cases who had associated anomalies (“LRD-multiples”) with isolated LRD cases for their associations with pre-gestational diabetes, maternal daily smoking and use of folate/ multivitamin supplements, the point estimates could suggest a closer association of daily smoking and pre-gestational diabetes with LRD-multiples than with isolated LRD. However, since we had to exclude TOPFAs, the number of multiple cases was very small, and confidence intervals were wide and overlapping. We were therefore unable to draw conclusions. Daily smoking was not, however, associated with isolated LRD, in contrast to pre-gestational diabetes, where statistically significant associations were found with both LRD-multiples and isolated LRD. Hyperglycemia is a well-known teratogenic factor for a wide range of congenital anomalies [24–26]. For use of folate/multivitamin supplements, the point estimates were quite similar for LRD-multiples and isolated LRD.

The present study was based on data from the MBRN alone. While information on some risk factors and life style habits are available in the registry, such as multivitamin/folate supplements and maternal smoking habits (both since 1999), others are not. Factors like maternal alcohol use or occupational exposures are not registered in the MBRN, and we were therefore unable to evaluate their possible associations with LRD.

We found increased risk of both stillbirth, neonatal death and infant death associated with LRD over the full time period. However, much of this increased mortality is likely related to the high proportion of associated anomalies. When excluding all cases with associated non-limb anomalies and looking only at the last time period where anomalies were better registered, there was no increased mortality risk associated with LRD. However, due to the very few deaths overall, this result must be interpreted with caution. The point estimate for stillbirth was compatible with a possible doubled risk associated with LRD although confidence intervals included 1.0, and we had inadequate power to disclose a potential true increase in risk of stillbirth.

### Strengths and limitations

Our aim was to review all LRD cases, 1970–1998, and recode from ICD-8 and internal codes to the more specific ICD-10, and to evaluate the results by comparing with published literature. The results for the recoded LRD group are mainly in agreement with the literature, and the MBRN now has ICD-10 codes for LRD cases back to 1970, a time when this version of the ICD was not yet in use. The MBRN is a nation-wide, population based registry with compulsory notification of all births in Norway since 1967, including late fetal losses, stillbirths and, since 1999, TOPFAs. Also since 1999, the registry has additionally received compulsory notification from NICUs for all infants transferred to such a unit after birth, and both these changes (TOPFA reports and NICU notifications) have led to a better ascertainment of congenital anomalies after 1998. Although LRD are rare congenital anomalies, we had enough data to look at specific limb defects, associated anomalies, and risk factors. For mortality we had enough data when looking at all LRD cases over the full study period, but when excluding associated non-limb anomalies and using data only after 1998, we lacked power to draw precise conclusions. Because notification to the registry is compulsory, selection bias is not a problem. The major limitation is that the information concerning LRD was often quite limited, especially before 1999 and especially concerning defects in the hands and feet, and we were therefore not able to classify the LRD cases according to established classification systems. The information on associated anomalies was also limited before 1999, making the years from 1999 the most valid for detailed analyses. For data from 1999, there was a relatively large proportion of missing information on maternal smoking habits and use of folate/multivitamin supplements, which we handled by multiple imputation after excluding TOPFAs. It is unfortunate that TOPFA cases did not have adequate information on maternal smoking, folate supplementation use or maternal chronic diseases.

We conclude that the MBRN now has information on LRD coded by ICD-10 from 1970, but that the information is not detailed enough to use established limb classification systems. Since aetiology may vary between the specific groups of LRD, we recommend that the registry collects radiographic descriptions or more detailed information from hospital records for these rare congenital anomalies. With a total prevalence at birth of less than 5 per 10 000 and a likely number of annual cases around 25–30 per year, we hope this is achievable. A prospective medical quality and research registry on all CULA cases was recently established in Division of Orthopaedic Surgery, Oslo University Hospital, which includes more specific and clinically relevant details on cases. This will be a good supplement to the MBRN, providing exciting possibilities for future data linkage.

## Supporting information

S1 Table Distribution of specific reduction defects in the two time periods (1970–1998: N = 706 cases, and 1999–2016: N = 500 cases), and for the whole study period (1970–2016, N = 1206 case). The Medical Birth Registry of Norway, 1970–2016.
(DOCX)

## References

[1] Vargesson N. Thalidomide-induced teratogenesis: history and mechanisms. Birth Defects Res C Embryo Today. 2015;105(2):140–56. 10.1002/bdrc.21096 26043938 PMC4737249

[2] Irgens LM. The Medical Birth Registry of Norway. Epidemiological research and surveillance throughout 30 years. Acta Obstet Gynecol Scand. 2000;79(6):435–9. 10857866

[3] The Personal Health Data Filing System Act https://lovdatano/dokument/NL/lov/2014-06-20-43. 2014.

[4] The Medical Birth Registry Regulation. https://lovdatano/dokument/SF/forskrift/2001-12-21-1483. 2001.

[5] Stoll C, Mastroiacovo P, de Wals P, Weatherall J, Garne E. Guide For the Description and Classification of Congenital Limb Defects http://wwweurocat-networkeu/content/EUROCAT-Guide-3pdf. 2004.

[6] Stoll C, Duboule D, Holmes LB, Spranger J. Classification of limb defects. Am J Med Genet. 1998;77(5):439–41. 9632177

[7] EUROCAT Subgroups of Congenital Anomalies. http://wwweurocat-networkeu/content/Section%2033-%2027_Oct2016pdf. 2014;EUROCAT Guide 1.4(Section 3.3).

[8] Khoury MJ, Botto L, Mastroiacovo P, Skjaerven R, Castilla E, Erickson JD. Monitoring for multiple congenital anomalies: an international perspective. Epidemiol Rev. 1994;16(2):335–50. 10.1093/oxfordjournals.epirev.a036157 7713183

[9] Garne E, Dolk H, Loane M, Wellesley D, Barisic I, Calzolari E, et al Paper 5: Surveillance of multiple congenital anomalies: implementation of a computer algorithm in European registers for classification of cases. Birth Defects Res A Clin Mol Teratol. 2011;91 Suppl 1:S44–50. 21384529 10.1002/bdra.20777

[10] Morris JK, Springett AL, Greenlees R, Loane M, Addor MC, Arriola L, et al Trends in congenital anomalies in Europe from 1980 to 2012. PLoS One. 2018;13(4):e0194986 10.1371/journal.pone.0194986 29621304 PMC5886482

[11] Firth HV, Boyd PA, Chamberlain P, MacKenzie IZ, Lindenbaum RH, Huson SM. Severe limb abnormalities after chorion villus sampling at 56–66 days' gestation. Lancet. 1991;337(8744):762–3. 10.1016/0140-6736(91)91374-4 1672394

[12] Bedard T, Lowry RB, Sibbald B, Kiefer GN, Metcalfe A. Congenital limb deficiencies in Alberta-a review of 33 years (1980–2012) from the Alberta Congenital Anomalies Surveillance System (ACASS). Am J Med Genet A. 2015;167A(11):2599–609. 10.1002/ajmg.a.37240 26171959

[13] Koskimies E, Lindfors N, Gissler M, Peltonen J, Nietosvaara Y. Congenital upper limb deficiencies and associated malformations in Finland: a population-based study. J Hand Surg Am. 2011;36(6):1058–65. 10.1016/j.jhsa.2011.03.015 21601997

[14] Ekblom AG, Laurell T, Arner M. Epidemiology of congenital upper limb anomalies in 562 children born in 1997 to 2007: a total population study from stockholm, sweden. J Hand Surg Am. 2010;35(11):1742–54. 10.1016/j.jhsa.2010.07.007 20961708

[15] Calzolari E, Manservigi D, Garani GP, Cocchi G, Magnani C, Milan M. Limb reduction defects in Emilia Romagna, Italy: epidemiological and genetic study in 173,109 consecutive births. J Med Genet. 1990;27(6):353–7. 10.1136/jmg.27.6.353 2359096 PMC1017130

[16] Froster-Iskenius UG, Baird PA. Limb reduction defects in over one million consecutive livebirths. Teratology. 1989;39(2):127–35. 10.1002/tera.1420390205 2784595

[17] Stoll C, Alembik Y, Dott B, Roth MP. Associated malformations in patients with limb reduction deficiencies. Eur J Med Genet. 2010;53(5):286–90. 10.1016/j.ejmg.2010.07.012 20670696

[18] Evans JA, Vitez M, Czeizel A. Congenital abnormalities associated with limb deficiency defects: a population study based on cases from the Hungarian Congenital Malformation Registry (1975–1984). Am J Med Genet. 1994;49(1):52–66. 10.1002/ajmg.1320490111 8172251

[19] Bedard T, Lowry RB, Sibbald B, Crawford S, Kiefer GN. Congenital limb deficiencies and major associated anomalies in Alberta for the years 1980–2012. Am J Med Genet A. 2018;176(1):19–28. 10.1002/ajmg.a.38513 29168277

[20] Holmes LB. Teratogen-induced limb defects. Am J Med Genet. 2002;112(3):297–303. 10.1002/ajmg.10781 12357474

[21] Holmes LB, Harvey EA, Coull BA, Huntington KB, Khoshbin S, Hayes AM, et al The teratogenicity of anticonvulsant drugs. N Engl J Med. 2001;344(15):1132–8. 10.1056/NEJM200104123441504 11297704

[22] Jentink J, Loane MA, Dolk H, Barisic I, Garne E, Morris JK, et al Valproic acid monotherapy in pregnancy and major congenital malformations. N Engl J Med. 2010;362(23):2185–93. 10.1056/NEJMoa0907328 20558369

[23] Froster UG, Baird PA. Maternal factors, medications, and drug exposure in congenital limb reduction defects. Environ Health Perspect. 1993;101 Suppl 3:269–74. 10.1289/ehp.93101s3269 PMC1521174 8143629

[24] Aberg A, Westbom L, Kallen B. Congenital malformations among infants whose mothers had gestational diabetes or preexisting diabetes. Early Hum Dev. 2001;61(2):85–95. 11223271 10.1016/s0378-3782(00)00125-0

[25] Taylor R, Davison JM. Type 1 diabetes and pregnancy. BMJ. 2007;334(7596):742–5. 10.1136/bmj.39154.700417.BE 17413175 PMC1847857

[26] Garne E, Loane M, Dolk H, Barisic I, Addor MC, Arriola L, et al Spectrum of congenital anomalies in pregnancies with pregestational diabetes. Birth Defects Res A Clin Mol Teratol. 2012;94(3):134–40. 10.1002/bdra.22886 22371321

[27] Czeizel A, Kodaj I, Lenz W. Smoking during pregnancy and congenital limb deficiency. BMJ. 1994;308:1473–6. 10.1136/bmj.308.6942.1473 8019281 PMC2540321

[28] Kallen K. Maternal smoking during pregnancy and limb reduction malformations in Sweden. Am J Public Health. 1997;87(1):29–32. 10.2105/ajph.87.1.29 9065222 PMC1380760

[29] Kallen K. Maternal malformations and maternal smoking Paediatr Perinat Epidemiol. 2000;14:227–33. 10949214 10.1046/j.1365-3016.2000.00269.x

[30] Aro T. Maternal diseases, alcohol consumption and smoking during pregnany associated with reduction limb defects. Early Hum Dev. 1983;9:49–57. 6667650 10.1016/0378-3782(83)90101-9

[31] Hackshaw A, Rodeck C, Boniface S. Maternal smoking in pregnancy and birth defects: a systematic review based on 173 687 malformed cases and 11.7 million controls. Hum Reprod Update. 2011;17(5):589–604. 10.1093/humupd/dmr022 21747128 PMC3156888

[32] Shaw GM, O'Malley CD, Wasserman CR, Tolarova MM, Lammer EJ. Maternal periconceptional use of multivitamins and reduced risk for conotruncal heart defects and limb deficiencies among offspring. Am J Med Genet. 1995;59(4):536–45. 10.1002/ajmg.1320590428 8585581

[33] Canfield MA, Collins JS, Botto LD, Williams LJ, Mai CT, Kirby RS, et al Changes in the birth prevalence of selected birth defects after grain fortification with folic acid in the United States: findings from a multi-state population-based study. Birth Defects Res A Clin Mol Teratol. 2005;73(10):679–89. 10.1002/bdra.20210 16240378

[34] Czeizel AE. Periconceptional folic acid containing multivitamin supplementation. Eur J Obstet Gynecol Reprod Biol. 1998;78(2):151–61. 9622312 10.1016/s0301-2115(98)00061-x

[35] Werler MM, Hayes C, Louik C, Shapiro S, Mitchell AA. Multivitamin supplementation and risk of birth defects. Am J Epidemiol. 1999;150(7):675–82. 10.1093/oxfordjournals.aje.a010070 10512421

[36] Wolf HT, Hegaard HK, Huusom LD, Pinborg AB. Multivitamin use and adverse birth outcomes in high-income countries: a systematic review and meta-analysis. Am J Obstet Gynecol. 2017;217(4):404 e1- e30. 10.1016/j.ajog.2017.03.029 28377269

[37] Cleves MA, Hobbs CA, Zhao W, Krakowiak PA, MacLeod SL, National Birth Defects Prevention S. Association between selected folate pathway polymorphisms and nonsyndromic limb reduction defects: a case-parental analysis. Paediatr Perinat Epidemiol. 2011;25(2):124–34. 10.1111/j.1365-3016.2010.01160.x 21281325 PMC3050483

[38] Bower C, Miller M, Payne J, Serna P. Folate intake and the primary prevention of non-neural birth defects. Aust N Z J Public Health. 2006;30(3):258–61. 16800203 10.1111/j.1467-842x.2006.tb00867.x

[39] Robitaille J, Carmichael SL, Shaw GM, Olney RS, National Birth Defects Prevention S. Maternal nutrient intake and risks for transverse and longitudinal limb deficiencies: data from the National Birth Defects Prevention Study, 1997–2003. Birth Defects Res A Clin Mol Teratol. 2009;85(9):773–9. 10.1002/bdra.20587 19350655

