# Supplementary material for: Epidemiology of limb reduction defects as registered in the Medical Birth Registry of Norway, 1970-2016: Population based study
Source: PLoS One. 2019 Jul 17;14(7):e0219930. doi: 10.1371/journal.pone.0219930 (PMC6636750; doi:10.1371/journal.pone.0219930)
Supplement: S1 Table — The Medical Birth Registry of Norway, 1970–2016. (DOCX) [file pone.0219930.s001.docx]

**S1 table: Distribution of specific reduction defects in the two time periods (1970-1998: N=706 cases, and 1999-2016: N=500 cases), and for the whole study period (1970-2016, N=1206 case). The Medical Birth Registry of Norway, 1970-2016.**

| ICD-10 Code | Text | 1970-1998,  N (%)^a^ | 1999-2016,  N (%)^a^ | 1970-2016,  N (%)^a^ |
| --- | --- | --- | --- | --- |
| Q710 | Congenital complete absence of upper limb(s) | 11 (1.6) | 10 (2.0) | 21 (1.7) |
| Q711 | Congenital absence of upper arm and forearm with hand present | 5 (0.7) | 4 (0.8) | 9 (0.7) |
| Q712 | Congenital absence of both forearm and hand | 81 (11.5) | 46 (9.2) | 127 (10.5) |
| Q713 | Congenital absence of hand and finger(s) | 261 (37.0) | 131 (26.2) | 392 (32.5) |
| Q714 | Longitudinal reduction defect of radius | 68 (9.6) | 74 (14.8) | 142 (11.8) |
| Q715 | Longitudinal reduction defect of ulna | 33 (4.7) | 8 (1.6) | 41 (3.4) |
| Q716 | Lobster-claw hand | 35 (5.0) | 19 (3.8) | 54 (4.5) |
| Q718 | Other reduction defects of upper limb(s) | 17 (2.4) | 73 (14.6) | 91 (7.5) |
| Q719 | Reduction defect of upper limb, unspecified | 32 (4.5) | 43 (8.6) | 75 (6.2) |
| Q720 | Congenital complete absence of lower limbs(s) | 15 (2.1) | 13 (2.6) | 28 (2.3) |
| Q721 | Congenital absence of thigh and lower leg with foot present | 3 (0.4) | 3 (0.6) | 6 (0.5) |
| Q722 | Congenital absence of both lower leg and foot | 9 (1.3) | 11 (2.2) | 20 (1.7) |
| Q723 | Congenital absence of foot and toe(s) | 96 (13.6) | 39 (7.8) | 135 (11.2) |
| Q724 | Longitudinal reduction defect of femur | 10 (1.4) | 19 (3.8) | 29 (2.4) |
| Q725 | Longitudinal reduction defect of tibia | 14 (2.0) | 15 (3.0) | 29 (2.4) |
| Q726 | Longitudinal reduction defect of fibula | 13 (1.8) | 10 (2.0) | 23 (1.9) |
| Q727 | Spilt foot | 14 (2.0) | 3 (0.6) | 17 (1.4) |
| Q728 | Other reduction defects of lower limb(s) | 18 (2.5 | 39 (7.8) | 57 (4.7) |
| Q729 | Reduction defect of lower limb, unspecified | 16 (2.3) | 26 (5.2) | 42 (3.5) |
| Q730 | Congenital absence of unspecified limb(s) | 1 (0.1) | 10 (2.0) | 11 (0.9) |
| Q731 | Phocomelia, unspecified limb(s) | 5 (0.7) | 5 (1.0) | 10 (0.8) |
| Q738 | Other reduction defects of unspecified limb(s) | 37 (5.2) | 18 (3.6) | 55 (4.6) |

^a^ The sum exceeds 100% as some cases have more than one defect.
